# Correlating Metal Poisoning with Zeolite Deactivation in an Individual Catalyst Particle by Chemical and Phase-Sensitive X-ray Microscopy**

**DOI:** 10.1002/anie.201210030

**Published:** 2013-04-25

**Authors:** Javier Ruiz-Martínez, Andrew M Beale, Upakul Deka, Mathew G O'Brien, Paul D Quinn, J Fred W Mosselmans, Bert M Weckhuysen

**Affiliations:** Inorganic Chemistry and Catalysis, Debye Institute for Nanomaterials Science, Utrecht University, Universiteitslaan 993584 CG Utrecht (The Netherlands) E-mail: b.m.weckhuysen@uu.nla.m.beale@uu.nl; Science Division, Diamond Light Source, Harwell Science and Innovation CampusDidcot, Oxon OX11 0DE (UK)

**Keywords:** catalyst deactivation, fluid catalytic cracking, heterogeneous catalysis, X-ray microscopy, zeolites

Chemical industries heavily rely on the use of heterogeneous catalysts. The development of more sustainable chemical processes requires, however, better catalyst formulations and ultimately tailoring of these catalytic materials for a specific application. A showcase example is fluid catalytic cracking (FCC), which is industrially applied to convert heavy oil fractions into more valuable chemicals, such as gasoline and olefins.^1^ The detrimental effect of metals originating from crude oil, especially Ni and V, on FCC catalysts is widely recognized.^2^ The metals damage the active zeolite phase, being either ultrastable zeolite Y (USY) or zeolite ZSM-5. As a result, pore accessibility and acidity are decreased, while dehydrogenation–hydrogenation reactions are favored, leading to increased coke formation. In the case of Ni, the main detrimental effect is coke formation,^3^ while V poisoning is associated with permanent zeolite damage in the presence of steam at high temperatures.2a

Several research groups have attempted to understand the mechanism of metal poisoning by observing the distribution of metals across the 50–150 μm FCC catalyst particle and between the different catalyst components; that is, zeolite, matrix (for example, alumina and clay), and additives, which comprise the FCC catalyst particle. Such characterization studies have been mostly conducted with invasive characterization methods.^4^ These investigations revealed that V is much more mobile than Ni and proceeds more quickly towards the interior of the FCC catalyst particle.4d

The mayor drawback with these previous studies is that they require an invasive preparation step, where the FCC catalyst particle is cut along the desired plane of analysis. This bisection is far from trivial and in most of the cases an alteration in the distribution of the distinct FCC components is observed. Therefore, a non-invasive approach can be expected to deliver more truthful information about the location of metal poisons. Furthermore, the relationship between metal poisoning and zeolite deactivation is not well understood, and to date there are no studies providing detailed information about the effect of deactivation on the crystalline zeolite structure within an individual FCC catalyst particle.

Herein we report for the first time the detrimental effect of metal poisons on the zeolitic material after deactivation in a commercial FCC unit at the level of a single catalyst particle. Using synchrotron-based hard X-ray radiation, the presence of Ni, V, as well as the crystalline phases can be determined with micrometre resolution in 2D or 3D. Furthermore, the non-invasive nature of the experimental approach avoids the pre-bisection of the FCC particle, avoiding damage and contamination to the catalyst material. Our findings lead to a better understanding of the deactivation processes taking place in real-life FCC catalysis and open the possibility to apply this approach for the study of other important catalytic materials, comprising both metals and crystalline phases.

Recent developments in synchrotron radiation now make it possible to image catalyst materials with high spatial resolution in a non-invasive fashion.^5^ Microfocus X-ray fluorescence (μ-XRF) microscopy is a powerful imaging technique widely applied in several disciplines.^6^ Furthermore, when combined with a scanning monochromator, it is possible to obtain microfocus X-ray absorption near-edge structure (μ-XANES) data so as to be able to obtain information on the oxidation and coordination state of the elements of interest. However, these methods fall short on giving information about the crystalline phases of the catalytic material; for example, the zeolite and clay phase. This can be done by using micro X-ray diffraction (μ-XRD), more in particular, XRD computed tomography (XRD-CT), which has been demonstrated recently in the field of heterogeneous catalysis to gain spatiotemporal insight into the active phase of a nickel supported γ-alumina catalyst body.^7^ Therefore, to accurately understand how metal poisons deactivate FCC catalysts at the single particle level and assess their effect on the local structure of the zeolite material embedded in the FCC matrix, the application of an integrated method comprising μ-XRF, μ-XANES, and μ-XRD is required in one set-up. This unique multipronged approach is illustrated in Figure 1; more details about the experimental approach can be found in the Supporting Information.

**Figure 1:**
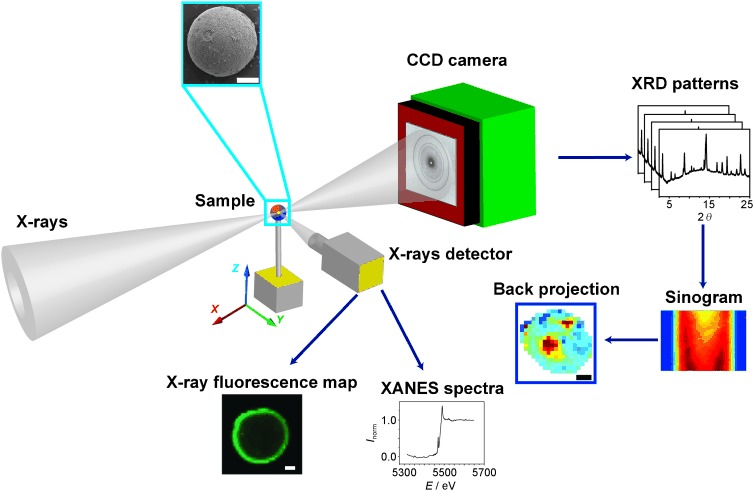
Investigating the deactivation of an individual catalyst particle mounted on a holder attached to a goniometer. μ-XRF and μ-XANES are collected by a polycapillary with confocal arrangement, which is placed on one side of the sample, while μ-XRD is measured in transmission mode by a 4000×2500 pixel CCD camera and subsequently radially integrated and transformed into 1D XRD patterns. The corresponding individual phases can be selected from the 1D XRD patterns and sinograms can be constructed. Finally, the sinograms can be back-projected, which provide the 2D maps of the corresponding crystalline phases present. Scale bars: 20 μm.

In a first step, 2D μ-XRF images of Ni and V were recorded on the FCC catalysts (Figure 2 a–f); 3D μ-XRF images were also acquired (Supporting Information, Movies S1 and S2). As Ni and V are not included in the catalyst formulation, no traces of these metals were observed in the fresh FCC sample (Figure 2 a and d). The same approach was applied for an equilibrium FCC catalyst (further denoted as Ecat), which is the fresh catalyst material after catalytic testing in an industrial cracking unit during real operation. In this sample, Ni and V were present with an uneven 2D distribution (Figure 2 b,c and e,f). A pronounced egg-shell profile was found for Ni with a shell thickness of 10–15 μm. Interestingly, V X-ray fluorescence was seen to span more evenly across the FCC particle, although its signal still strongly decreases in intensity with increasing probing depth within the particle. These results are in good agreement with previous invasive studies on FCC particles, which were bisected and metal profiles monitor with SIMS^**[**4c]^ and SEM-EDX.^8^ It should be noted that the Ni and V X-ray fluorescence on the right side of the FCC particle starts to fade, which is due to X-ray fluorescence attenuation. To ensure that such fluorescence attenuation does not affect the distribution of the metal poisons, an additional μ-XRF study was carried out. Identical μ-XRF measurements were performed on FCC catalysts particles impregnated with Ni and V, namely a Mitchell FCC catalyst particle. This lab-based deactivation method leads to an even Ni and V distribution across the FCC particle (Figure 2 c and f) and therefore the influence of X-ray attenuation can be accurately determined. More details on this reference experiment can be found in the Supporting information. 1D XRF intensity profiles (Figure 2 h and i) corroborate these findings.

**Figure 2:**
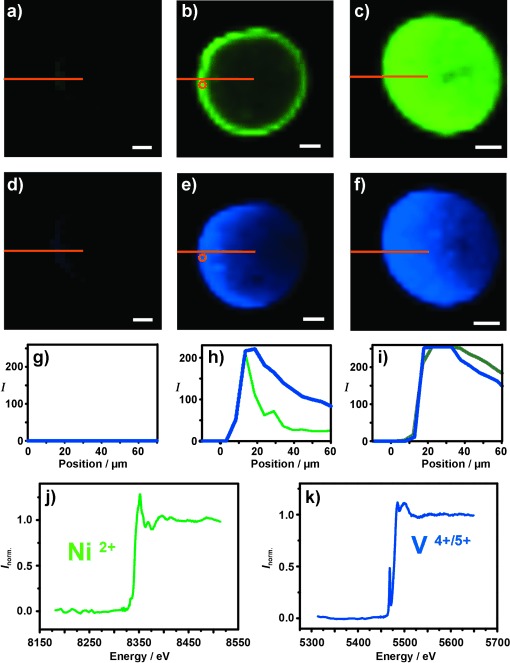
μ-XRF 2D chemical maps of Ni (a–c) and V (d–f) for a fresh (a,d), Ecat (b,e), and Mitchell (c,f) FCC catalyst particle. The orange lines illustrate the position where the intensity profiles were taken. Scale bars: 20 μm. g–i) 1D X-ray fluorescence intensity profiles as a function of the position inside the FCC catalyst particle derived from the 2D images for a g) fresh, h) Ecat, and i) Mitchell FCC catalyst particle. j) Ni and k) V μ-XANES spectra of the spots highlighted with the orange circle for the Ecat particle. The spatial resolution of μ-XRF maps and μ-XANES spectra was 5×5 μm^2^.

Ni and V K-edge XANES spectra of the Ecat sample were then collected and the results are given in Figure 2 j and k, respectively. The results derived from the first-derivative data reveal that the Ni K-edge is at 8343 eV, which is indicative for Ni^2+^.^9^ The small pre-edge at 8330.5 eV is attributed to the 1s–3d transition and suggests the presence of an oxide-type phase, most likely NiAl_2_O_4_ and/or NiO species in which the Ni^2+^ is six-coordinate.^10^ In the case of V, the XANES spectrum shows a K-edge at 5480.4 eV and a pre-edge peak at 5469.4 eV. The position of both features is indicative for a mixture of V^4+^ and V^5+^. Furthermore, the intensity of the pre-edge peak implies that most likely there is a mixture of octahedral V_2_O_4_ and square-pyramidal V_2_O_5_ species.^11^ To validate our findings, an additional XANES study was performed where spectra from V_2_O_4_ and V_2_O_5_ reference compounds and bulk measurements of the Ecat sample were measured (Supporting Information, Figure S1).

μ-XRD-CT provides complementary information regarding the crystalline phases. Representative XRD patterns of both samples (Figure 3) display the sum of the diffraction patterns recorded for the acquired projections of the middle plane of fresh and an Ecat FCC catalyst particles. This represents an average of 2700 collected μ-XRD patterns per sample and allows the detection of all of the crystalline phases present. A number of reflections can be identified for the fresh FCC catalyst particle, which together represents all the FCC components with the exception of silica (amorphous). More specifically, the diffraction patterns of zeolite Y, kaolinite, and boehmite can be identified in the fresh FCC particle. An additional phase, anatase, is also detected, which most probably originates from TiO_2_ impurities in the kaolinite clay fraction. Interestingly, the summed XRD pattern of the Ecat sample is very different to that of the fresh sample. More specifically, the reflections corresponding to zeolite Y are less intense, which can be related to a decrease in the overall crystallinity of the zeolite material. Kaolinite, which has a layered structure, undergoes also a series of phase transformations resulting in the formation of a mullite phase. Boehmite dehydrates and subsequently forms a γ-alumina phase. Finally, no phases from the Ni and V could be detected, which suggest that they are below the detection limit or distributed as small oxide and aluminate nanoparticles.

**Figure 3:**
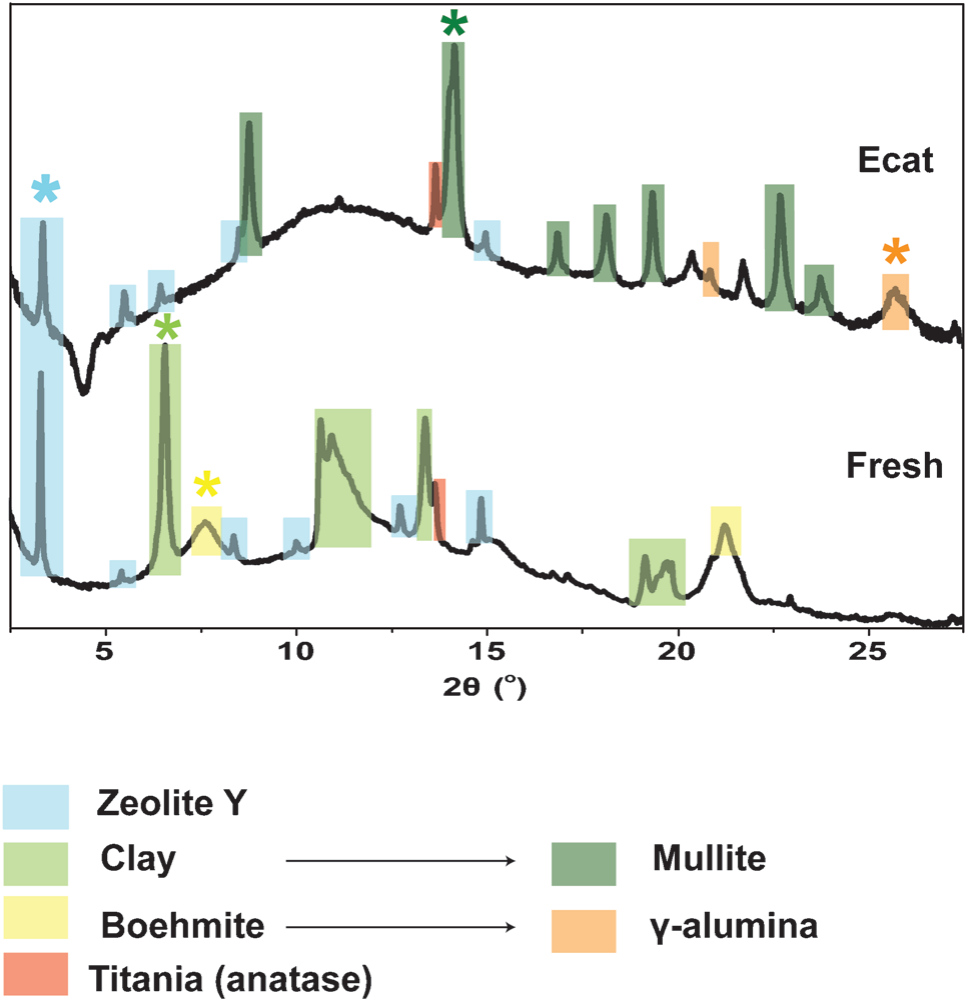
Summed XRD patterns of an individual fresh and Ecat FCC catalyst particle. The colored squares denote the characteristic reflections of the crystalline phases present; asterisks refer to the reflections selected for the 2D reconstruction of the crystalline phases.

These identified XRD-active phases can be also transformed into 2D distribution maps by using a reconstruction algorithm. More details about this mathematical procedure can be found in the Supporting information as well as in recent literature.^12^ Figure 4 a–f illustrate how this approach can discriminate between the different phases present and also spatially resolve them. Furthermore, the transformation of these crystalline components upon poisoning/deactivation can be assessed by using a specific diffraction peak per phase, indicated in the summed diffraction patterns by asterisks in Figure 3.

**Figure 4:**
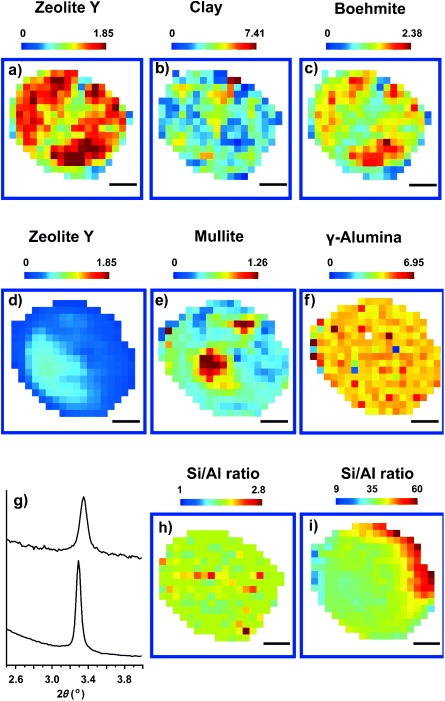
2D maps of a) zeolite Y, b) clay, and c) boehmite for a fresh FCC particle, and 2D maps of d) zeolite Y, e) mullite, and f) γ-alumina for the Ecat particle. g) Magnification of the (111) diffraction peak for the fresh (lower) and Ecat sample (upper) to evaluate the peak shift experienced after deactivation. 2D reconstructions of the zeolite Si/Al ratio for the h) fresh and i) Ecat samples; thermal scale bar shows the position of the diffraction peak for the fresh and the calculated zeolite Si/Al ratio for the Ecat FCC catalyst particle. Scale bars: 20 μm.

We now focus our attention on the physicochemical changes taking place in zeolite Y upon deactivation/poisoning. By scanning the whole FCC particle 2D zeolite Y maps can be generated from the most intense diffraction peak at 2*θ*≍3.3°; that is, the (111) reflection. Figure 4 a reveals a random distribution of the zeolite component throughout the fresh FCC particle, which is in agreement with previous studies.^13^ Interestingly, after deactivation, the zeolite undergoes significant changes in crystallinity and spatial distribution, leading to a less ordered material, with a preferential destruction of the zeolite at the periphery of the particle, resulting in an egg-yolk distribution of the zeolitic material (Figure 4 d). Our previous results using confocal fluorescence microscopy and a fluorescent probe reaction that is very sensitive to the acidity of the zeolite were unable to monitor such a zeolitic distribution in the interior of the FCC catalyst bodies.13a This is most probably due to the attenuation suffered by the fluorescent light in the interior of the catalyst particle, which allows investigation of only the first 10–15 μm of the material in an accurate manner. More importantly, the egg-yolk distribution of the zeolite phase mirrors with the egg-shell distribution of the poisons in the Ecat sample, providing direct evidence for the negative effect of Ni and V on the crystallinity of the zeolite phase.

In a next step, we aimed for a better understanding of this 2D egg-yolk zeolite phase degradation by studying in more detail the obtained XRD patterns. Inspection of the (111) reflection (peak at 2*θ*≍3.3°, as depicted in Figure 4 g) and the (220) reflection (peak at 2*θ*≍5.4°; Supporting Figure S2a) reveals significant peak shifts at different locations in the FCC catalyst particle. To corroborate that these peak shifts are not just artefacts that are due for example to changes in the experimental setup geometry by a longer distance between the sample and the CCD camera detector, bulk XRD analysis with a standard powder X-ray diffractometer were performed (Supporting Information, Figure S3). However, this analysis rules out this hypothesis and confirms that the peak shifts are real. These peak shifts to higher 2*θ* values are well-described in the literature and indicate a shift towards a smaller d-spacing and reduced unit cell parameters of the zeolite, as a result of partial zeolite dealumination.^14^ As a consequence of this dealumination process, the zeolite experiences a loss in Brønsted acidity, which is one of the main causes of catalyst deactivation.^15^ Therefore, our approach allows an estimation of the extent of dealumination, which can be represented in the form of a 2D map of Si/Al ratios across an Ecat FCC catalyst particle (Figure 4 i).

Although a narrow range of peak positions is evenly distributed for the fresh FCC catalyst particle (Figure 4 h), the Ecat FCC particle shows a shift in 2*θ* positions to higher 2*θ* values, more specifically in the periphery, which can be translated into a higher degree of zeolite dealumination in the outer rim of the particle. Translating the peak shifts into Si/Al ratios is possible by making use of a proper calibration curve (Supporting Information, Figure S4). A unit cell analysis of bulk powder XRD patterns was performed for zeolite Y samples with known Si/Al ratios. Furthermore, the same was carried out for fresh catalyst particles, which were deactivated under distinct steaming conditions. The corresponding d-values from the samples were then used to estimate a particular Si/Al ratio for the Ecat samples under study. The fresh sample shows a range of Si/Al ratio, which is in line with the values that the catalyst manufacturer employs for the synthesis of the catalyst particles. The unequal spatial zeolite destruction and related dealumination of a single FCC catalyst particle (Figure 4 i) is very significant as the Si/Al ratios range from about 60 at the periphery to circa 35 in the interior of the Ecat FCC catalyst particle. Furthermore, it has to our best knowledge never been reported and can be explained in two ways. A first plausible explanation is that Ni and V, in combination with high boiling point hydrocarbons, are unable to access the center of the particle and therefore results in significantly higher coke formation at the external part of the particle. During regeneration, coke combustion will create hot spots and therefore this localized severe hydrothermal condition will have a higher impact on the zeolite destruction and related dealumination. A second reasoning relates to the egg-shell distribution of V, which promotes zeolite destruction by mechanisms described in the literature.2a

In conclusion, the developed combined μ-XRF/μ-XANES/μ-XRD approach is capable of revealing 2D and 3D chemical information in commercially used FCC catalyst materials at the individual particle level. μ-XRF revealed in an Ecat particle egg-shell distributions for V and Ni, while μ-XRD indicated for the same catalyst particle egg-yolk distributions for zeolite crystallinity and the Si/Al ratio, directly linking the detrimental effect of metal poisoning with zeolite destruction and dealumination. It is clear that this multipronged X-ray microscopy method offers great potential for the 2D and 3D spatiotemporal characterization of other catalyst materials containing metals and crystalline structures.

## Supplementary material

As a service to our authors and readers, this journal provides supporting information supplied by the authors. Such materials are peer reviewed and may be re-organized for online delivery, but are not copy-edited or typeset. Technical support issues arising from supporting information (other than missing files) should be addressed to the authors.

## References

[1] Cheng WC, Habib ET, Rajagopalan K, Roberie TG, Wormsbecher RF, Ziebarth MS, Ertl G, Knözinger H, Schüth F, Weitkamp J (2008). Handbook of Heterogeneous Catalysis, Vol. 6.

[2a] Trujillo CA, Navarro Uribe U, Knops-Gerrits PP, Oviedo LA, Jacobs PA (1997). J. Catal.

[2b] Xu M, Liu X, Madon RJ (2002). J. Catal.

[2c] Escobar AS, Pinto FV, Cerqueira HS, Pereira MM (2006). Appl. Catal. A.

[3] Cadet V, Raatz F, Lynch J, Marcilly C (1991). Appl. Catal.

[4a] Chao KJ, Lin LH, Ling YC, Hwang JF, Hou LY (1995). Appl. Catal. A.

[4b] Cao H, Suib SL (1995). Appl. Spectrosc.

[4c] Kugler EL, Leta DP (1988). J. Catal.

[4d] Lappas AA, Nalbandian L, Iatridis DK, Voutetakis SS, Vasalos IA (2001). Catal. Today.

[4e] Haas A, Suarez W, Young GW (1992). AIChE Symp. Ser.

[5a] Beale AM, Jacques SDM, Weckhuysen BM (2010). Chem. Soc. Rev.

[5b] Buurmans ILC, Weckhuysen BM (2012). Nat. Chem.

[5c] Weckhuysen BM (2009). Angew. Chem.

[b01] (2009). Angew. Chem. Int. Ed.

[5d] Grunwaldt JD, Wagner JB, Dunin-Borkowski RE (2012). ChemCatChem.

[5e] Grunwaldt JD, Schroer CG (2010). Chem. Soc. Rev.

[6a] Brugger J, Pring A, Reith F, Ryan C, Etschmann B, Liu W, O’Neill B, Ngothai Y (2010). Radiat. Phys. Chem.

[6b] Cotte M, Susini J, Metrich N, Moscato A, Gratziu C, Bertagnini A, Pagano M (2006). Anal. Chem.

[6c] Fahrni CJ (2007). Curr. Opin. Chem. Biol.

[6d] Basile F, Benito P, Bugani S, De Nolf W, Fornasari G, Janssens K, Morselli L, Scavetta E, Tonelli D, Vaccari A (2010). Adv. Funct. Mater.

[7a] Jacques SMD, Di Michiel M, Beale AM, Sochi T, O’Brien MG, Espinosa-Alonso L, Weckhuysen BM, Barnes P (2011). Angew. Chem.

[b02] (2011). Angew. Chem. Int. Ed.

[7b] O’Brien MG, Jacques SDM, Di Michiel M, Barnes P, Weckhuysen BM, Beale AM (2012). Chem. Sci.

[8] Psarras AC, Iliopoulou EF, Nalbandian L, Lappas AA, Pouwels C (2007). Catal. Today.

[9a] Gardner TH, Spivey JJ, Kugler EL, Campos A, Hissam JC, Roy AD (2010). J. Phys. Chem. C.

[9b] Woolley RJ, Illy BN, Ryan MP, Skinner SJ (2011). J. Mater. Chem.

[10] Beale AM, Paul M, Sankar G, Oldman RJ, Catlow CRA, French S, Fowles M (2009). J. Mater. Chem.

[11] Chaurand P, Rose J, Briois V, Salome M, Proux O, Nassif V, Olivi L, Susini J, Hazemann J-L, Bottero J-Y (2007). J. Phys. Chem. B.

[12] Bleuet P, Welcomme E, Dooryhée E, Susini J, Hodeau JL, Walter P (2008). Nat. Mater.

[13a] Buurmans ILC, Ruiz-Martínez J, Knowles WV, van der Beek D, Bergwerff JA, Vogt ETC, Weckhuysen BM (2011). Nat. Chem.

[13b] Buurmans ILC, Ruiz-Martínez J, Van Leeuwen SL, Van Der Beek D, Bergwerff JA, Knowles WV, Vogt ETC, Weckhuysen BM (2012). Chem. Eur. J.

[13c] Ruiz-Martínez J, Buurmans ILC, Knowles WV, Van Der Beek D, Bergwerff JA, Vogt ETC, Weckhuysen BM (2012). Appl. Catal. A.

[14] Ray GJ, Meyers BL, Marshall CL (1987). Zeolites.

[15a] Vermeiren W, Gilson JP (2009). Top. Catal.

[15b] Newsam JM (1986). Science.

[15c] Corma A (1995). Chem. Rev.

[15d] Balmoos Rvon, Harris DH, Magee JS, Ertl G, Knözinger H, Weitkamp J (1997). Handbook of Heterogeneous Catalysis.

