# Supplementary material for: Correlating Metal Poisoning with Zeolite Deactivation in an Individual Catalyst Particle by Chemical and Phase-Sensitive X-ray Microscopy
Source: Angew Chem Int Ed Engl. 2013 Apr 25;52(23):5983–7. doi: 10.1002/anie.201210030 (PMC3749464; doi:10.1002/anie.201210030)
Supplement: Supplementary file 1 [file anie0052-5983-SD1.pdf]

Angewandte Chemie
Eine Zeitschrift der Gesellschaft Deutscher Chemiker

Supporting Information

© Wiley-VCH 2013

69451 Weinheim, Germany

Correlating Metal Poisoning with Zeolite Deactivation in an Individual Catalyst Particle by Chemical and Phase-Sensitive X-ray Microscopy**

Javier Ruiz-Martínez, Andrew M. Beale, Upakul Deka, Mathew G. O'Brien, Paul D. Quinn, J. Fred W. Mosselmans, and Bert M. Weckhuysen**

anie_201210030_sm_miscellaneous_information.pdf
anie_201210030_sm_ni_stack_movie.avi
anie_201210030_sm_v_stack_movie.avi

Supporting Information

Experimental Section

A single FCC catalyst particle is mounted in a sample holder, which consists of a stainless steel rod with a tip made of X-ray transparent material (i.e., kapton). Then the sample holder is mounted on a goniometer connected to the X-ray-imaging beamline I18 at the Diamond light source. The 10 keV monochromatic beam is focused on the sample by Kirkpatrick-Baez mirrors down to a spot size of 5 × 5 μm. The XRF and XANES data were collected using polycapillary microfocus lenses, whereas transmission XRD data were acquired by using a 4000 x 2500 pixel CCD camera.

3D μ-XRF images

The acquisition of the 3-D images was performed by staking 2-D images with ImageJ software. The stacks of the nickel and vanadium μ-XRF single images are represented as a movie in Supplementary video 1 and 2.

Ni Stack movie.avi V Stack movie.avi

XANES experiments

XANES spectra of the bulk Ecat, as well as VO_2 (Aldrich) and V_2O_5 (Acros) were taken at the DUBBLE (BM26A) beamline of the European Synchrotron Research Facility (ESRF, Grenoble, France). Measurements were taken in fluorescence mode in a glass capillary.

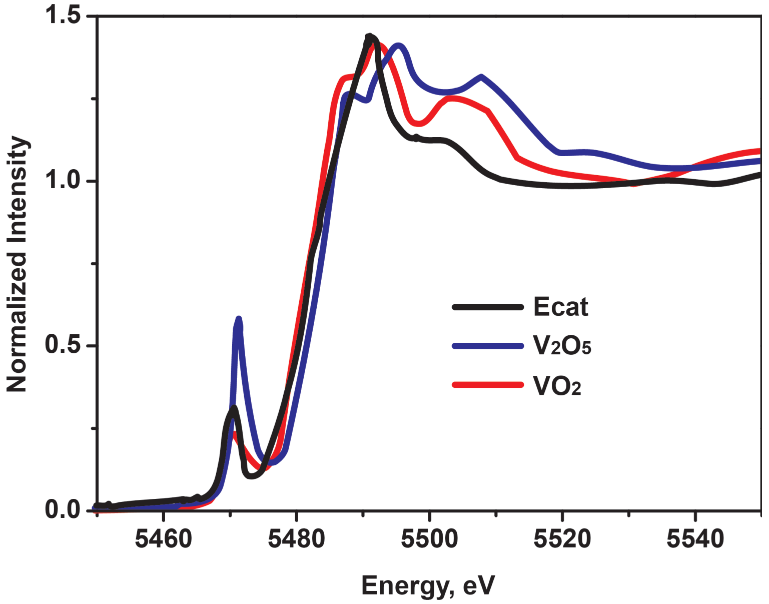

Figure S1. K-edge XANES bulk measurements of the Ecat samples (black line), as well as VO_2 (red line) and V_2O_5 (blue line) reference spectra.

XRD data acquisition and tomographic reconstruction

For each sample 90 rotations and 30 positions were measured, i.e., 2700 XRD patterns. The 2-D diffraction patterns collected by the CCD camera are integrated over the azimuthal angle and converted into the respective 1-D diffraction patterns. In order to identify all the crystalline phases, a 1-D sum diffraction pattern is calculated by summing the 2700 projections. The 1-D diffraction patterns were integrated along 2θ, and this was plotted as a function of the position and the rotation to build up the global sinogram, which contains the information of the diffraction information of the overall 2-D section. Then by selecting a representative diffraction peak, a sinogram of each crystalline phase was defined. Finally, a back projection of these sinograms provides the corresponding phase distribution maps.

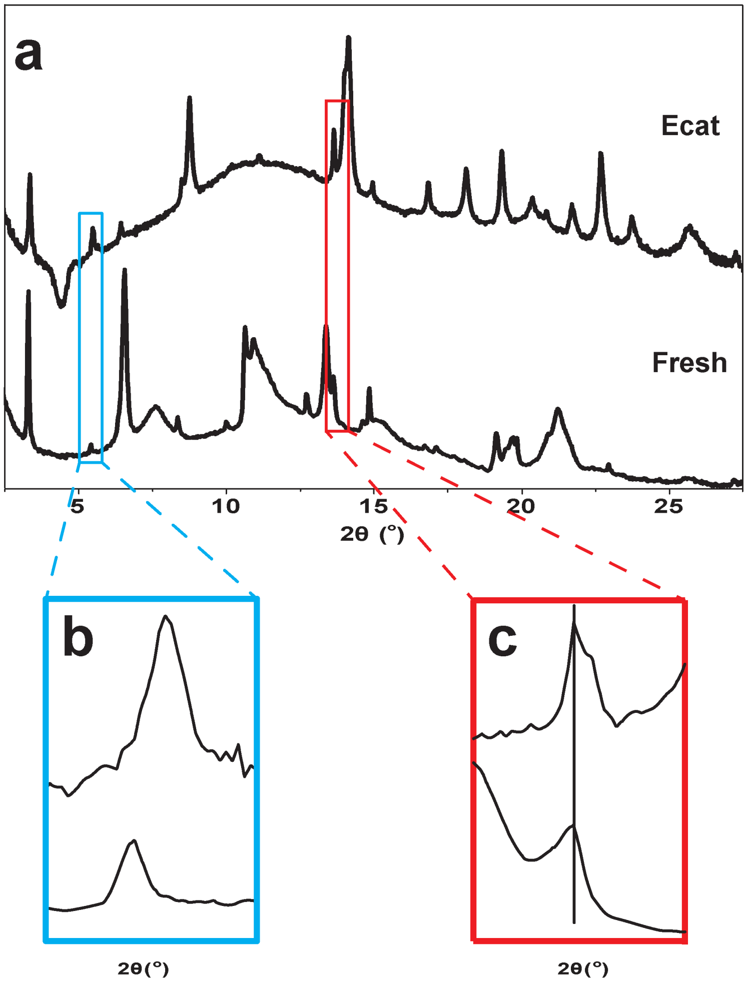

Figure S2. (a) Summed XRD patterns of a single fresh and Ecat FCC catalyst particle. (b) Magnification of the (220) diffraction peak of the zeolitic material from the summed XRD patterns of a single fresh and Ecat catalyst particle in order to evaluate the peak shift experienced after deactivation. (c) Magnification of the (101) diffraction peak of anatase for the fresh and Ecat catalyst particles. The anatase peak, which can be used as an internal standard, maintains its position in both samples and therefore verify that the shifts observed in the zeolite material are real.

X-ray powder diffraction

X-ray powder diffraction patterns of the ground FCC catalyst particles were acquired using a Bruker-AXS D8 Advance powder X-ray diffractometer. The diffractometer was equipped with an automatic divergence slit, a Våntec-1 detector, and a cobalt K-alpha 1,2 (λ = 1.79026 Å) source. Diffraction patterns were collected between 4-70 2θ with an increment of 0.017 (2θ) and an acquisition time of 2 s per step for the fresh and 4 s per step for the MI and Ecat samples. Supplementary Figure S1 shows the XRD powder patterns for the fresh and Ecat FCC catalyst particles. Phase assignment is based on reference patterns from the ICSD. Five crystalline phases are identified: zeolite Y (PDF 39-1380), kaolinite (PDF 14-0164), boehmite (PDF 21-1307), anatase TiO_2 (PDF 21-1272) and mullite (PDF 83-1881).

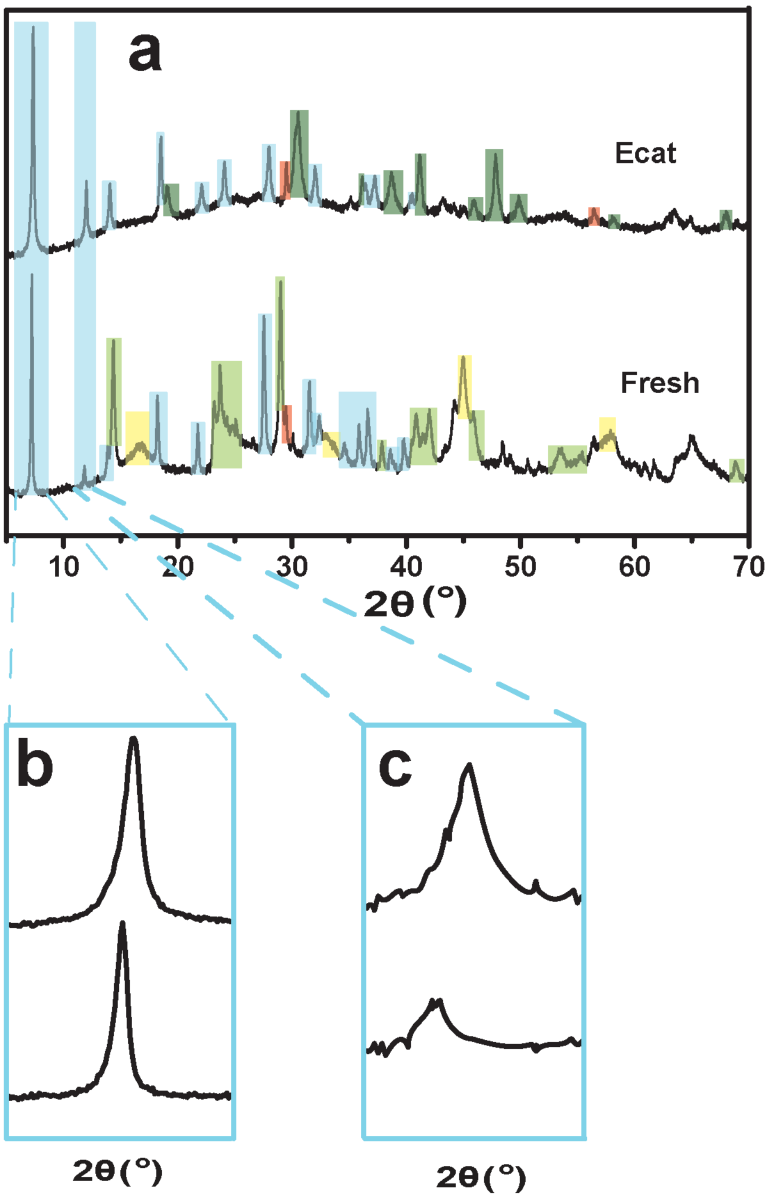

Figure S3. (a) X-ray diffraction powder patterns the fresh FCC catalyst and the Ecat. Coloured boxes indicate the reflections of the zeolite Y (blue), kaolinite (light green), boehmite (yellow), TiO_2 in anatase phase (red) and mullite (dark green). Magnification of the (b) (111) and (c) (220) diffraction peaks of the zeolitic material.

Peaks at ~3.3, 5.5, 8.5, 10.5, 13.0, and 14.9 correspond to the zeolite Y. Peaks at ~6.6, 10.6, 10.9, 13.4, 19.2, 19.7 and 19.8 correspond to kaolinite. Peaks at ~7.6 and 21.2 correspond to Al_2O_3 in boehmite phase. Peak at ~13.6 corresponds to TiO_2 in anatase phase. Peaks at ~8.8, 14.0, 14.2, 16.9, 18.1, 19.4, 22.7 and 23.7 correspond to mullite.

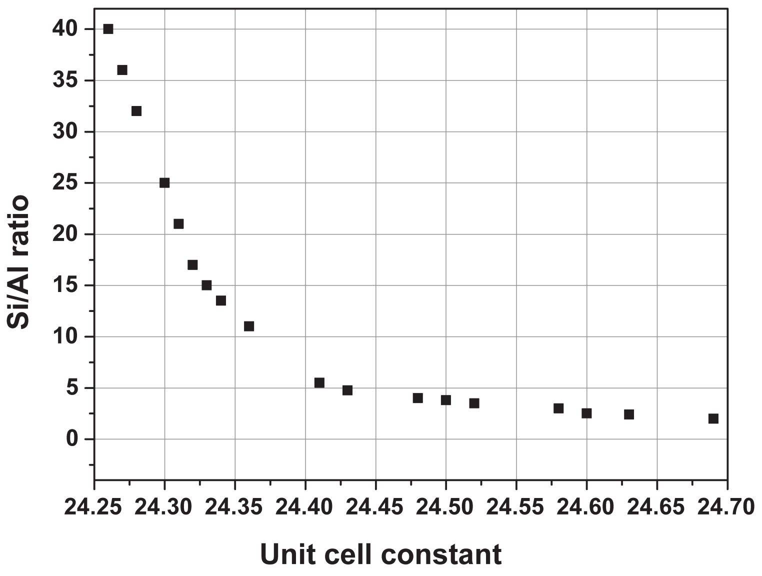

Figure S4. Calibration curve for the translation of the peak shifts into Si/Al ratios.
